# Three-Dimensional Liver Culture Systems to Maintain Primary Hepatic Properties for Toxicological Analysis In Vitro

**DOI:** 10.3390/ijms221910214

**Published:** 2021-09-23

**Authors:** Sarah Kammerer

**Affiliations:** Institute of Biotechnology, Brandenburg University of Technology Cottbus-Senftenberg, Universitätsplatz 1, 01968 Senftenberg, Germany; sarah.kammerer@b-tu.de; Tel.: +49-3573-85-934

**Keywords:** hepatocytes, HepG2, HepaRG, upcyte hepatocytes, 3D culture systems, primary hepatic function, toxicology, spheroids, perfused bioreactors, liver-on-a-chip

## Abstract

Drug-induced liver injury (DILI) is the major reason for failures in drug development and withdrawal of approved drugs from the market. Two-dimensional cultures of hepatocytes often fail to reliably predict DILI: hepatoma cell lines such as HepG2 do not reflect important primary-like hepatic properties and primary human hepatocytes (pHHs) dedifferentiate quickly in vitro and are, therefore, not suitable for long-term toxicity studies. More predictive liver in vitro models are urgently required in drug development and compound safety evaluation. This review discusses available human hepatic cell types for in vitro toxicology analysis and their usage in established and emerging three-dimensional (3D) culture systems. Generally, 3D cultures maintain or improve primary hepatic functions (including expression of drug-metabolizing enzymes) of different liver cells for several weeks of culture, thus allowing long-term and repeated-dose toxicity studies. Spheroid cultures of pHHs have been comprehensively tested, but also other cell types such as HepaRG benefit from 3D culture systems. Emerging 3D culture techniques include usage of induced pluripotent stem-cell-derived hepatocytes and primary-like upcyte cells, as well as advanced culture techniques such as microfluidic liver-on-a-chip models. In-depth characterization of existing and emerging 3D hepatocyte technologies is indispensable for successful implementation of such systems in toxicological analysis.

## 1. Introduction

Any organotypic in vitro model becomes obsolete if it does not represent the unique characteristics of the tissue of origin in the focus of the research. In the field of toxicological studies on liver cells, it is a prerequisite that hepatocytes cultured in vitro display features of their in vivo counterparts. This is a major challenge, as primary hepatocytes dedifferentiate very quickly after isolation and culture. The downregulation of hepatic properties already begins within 30 min of classical monolayer (two-dimensional; 2D) culture; within hours, crucial features such as the expression of albumin and cytochrome P450 (CYP) enzymes are almost lost, and typical hepatic morphology is deteriorated from day 2–3 onward. It has been shown that these dramatic changes are linked to an altered expression of miRNAs that downregulate those genes of interest and that also rearrange the lipid metabolism [1,2]. Primary human hepatocytes (pHHs), thus, only provide the possibility of short-term (acute) toxicological analysis. Other methods, cells and/or culture conditions must be used for standardized long-term (chronic) in vitro research on the effects of toxic compounds.

Such long-term analyses of potentially liver-toxic substances are however indispensable in drug development. Drug-induced liver injury (DILI) is the major reason for failure of drug development as well as for drug withdrawal from the market after initial approval [3,4]. In addition, 13% of acute liver failures and about 15% of all liver transplantations are caused by DILI [5,6]. Since 1990, 14 compounds were withdrawn from the market due to hepatic toxicities [7]. This number might seem small at first glance, but the economic damage and patients’ suffering behind the number are immense. Therefore, toxicity profiles of newly developed compounds must be detected as early as possible in preclinical, i.e., before first-in-men, trials. Further, it is also well known that animal models do not accurately reflect the human situation, as the expression of genes involved in drug absorption, distribution, metabolism, and excretion (ADME) differs considerably between species [8].

Thus, there is an urgent need for reliable, stable, and reproducible in vitro models that reflect physiological characteristics of the human liver for drug development and especially toxicity studies. This review will give an overview of different human hepatic cell systems and how they perform in established and emerging three-dimensional (3D) culture techniques. Three-dimensional culture techniques of hepatocytes generally aim to maintain and/or improve primary hepatic properties and, therefore, provide not only well-accepted, but also promising tools for application in toxicology analyses.

## 2. Human Hepatocyte In Vitro Models

Three-dimensional culture systems for human hepatocytes have become a valuable tool to study toxicity profiles of newly developed drugs. For example, spheroid cultures have been well known for decades, while other techniques have just recently developed. Apart from the different 3D culture techniques, it is also crucial to choose an appropriate cellular model and medium composition. With the intention to perform toxicity studies on such hepatic cells, it is important that they reflect the phenotype of the human liver in vivo. Among others, the following features of hepatocytes are of special interest:

- Albumin synthesis, urea production, and glycogen storage
- Expression of phase I enzymes involved in drug metabolism (CYP1A2, CYP2B6, CYP2C9, CYP2C19, CYP2D6, CYP3A4)
- Expression of phase II enzymes such as UDP-glucuronyl transferases (UGTs), glutathione-S-transferases (GSTs) or sulfotransferases (SULTs)
- Cellular polarization marked by the expression of F-actin and transporter molecules (also referred to as phase III) such as MRP2, Pgp, BSEP, OCT1, or OATP1

**Figure 1:**
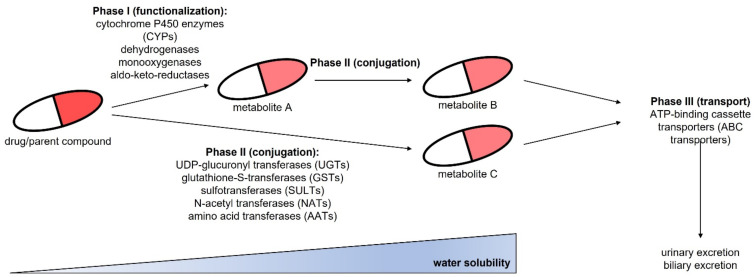
Overview of hepatic drug metabolism and excretion via phase I, phase II and phase III reactions.

Hepatic drug metabolism by phase I, II and III enzymes is depicted in Figure 1. Phase I enzymes are responsible for functionalization of compounds, while phase II enzymes perform conjugation reactions. During these metabolic changes, the water solubility of the drug increases. This allows drug clearance via the biliary or urinary excretion paths after metabolite export from hepatic cells via phase III transporters to the blood or bile. All these molecules, and especially members of phase III, might be involved in drug resistance. Consequently, particularly in long-term or repeated-dose hepatotoxicity studies, it might be of interest to also observe changes in such markers of hepatic drug resistance. For example, it was shown that hepatocytes become resistant to acetaminophen via increased expression of MRP3 and MRP4 transporters [9].

Besides the difficulty of choosing a suitable cellular model, it is also crucial to select the appropriate culture medium. Many vendors offer cell media that are designed to maintain or to improve hepatic properties of primary or other hepatic cells. However, those commercially available media often do not disclose all ingredients. This incomplete information makes it difficult to correctly interpret the results. The same holds true when serum-supplemented medium is used. Serum might improve viability and performance of the cells, but information is missing regarding its constitution. Therefore, the use of chemically-defined culture medium without serum should be recommended for toxicology analyses on hepatocytes.

The following sections discuss characteristics, as well as advantages and disadvantages of available liver cell models when they are cultured in standard monolayer format. 

### 2.1. Primary Human Hepatocytes

Freshly isolated or cryopreserved primary human hepatocytes (pHHs) are regarded as gold standard for toxicity analyses as they reflect the properties and functions of liver cells in vivo. Their major drawback is the above-mentioned, fast dedifferentiation occurring as soon as they are cultured in vitro in 2D [2]. Their lack of proliferation competency adds another difficulty, as the limited availability of cells from the same donor might not allow for repeated experiments or for the choice of larger experimental setups. PHHs can be obtained commercially or via collaborations with university clinics or hospitals. It is the nature of pHHs to be highly donor specific, as genetic setups might vary greatly between individuals [10,11]. This can be regarded as disadvantage, as standardization is almost impossible with such high interindividual differences. On the other hand, these variabilities allow us to perform analyses on cells with different genetic background and on polymorphisms, which are of special interest in detecting DILI.

### 2.2. HepG2 Cells

Established cell lines such as HepG2 are commonly used for toxicological studies as they are easy to handle, limitless available and cheap in maintenance. Further, a huge number of studies is available on those cells which makes it easy to compare new results to existing data. HepG2 cells were derived from a hepatoblastoma of a 15-year-old Caucasian male [12,13]. Despite their cancerous origin, HepG2 cells retained some primary characteristics, including the capacity of albumin synthesis [14] and glycogen storage [15]. However, they display only an extremely low to even absent expression of drug-metabolizing phase I (except CYP2B6) and phase II enzymes, which renders them a questionable tool for ADME studies [16,17]. To overcome this limitation, HepG2 cells have been genetically modified to stably overexpress one or more CYP enzymes [18,19,20,21,22]. This turns them into an adequate tool for studying the involvement of the respective CYP(s) in liver toxicity.

### 2.3. HepaRG Cells

In recent years, the HepaRG cell line [23] has gained increasing interest. The cell line was derived from a liver tumor but displays hepatocyte-like features after a differentiation procedure. HepaRG cells show activities of several phase I and phase II biotransformation enzymes, even in the range of freshly isolated pHHs [16,24,25]. As other tumor cell lines, HepaRG is easy to handle, and allows for long-term culture and repeated-dose experiments [26]. Apart from the fact that HepaRG are tumor cells, they have the disadvantages that they need a complex and time-consuming differentiation procedure and that they represent only one single donor. This is common to all cell lines, but the donor of HepaRG was a poor metabolizer for CYP2D6, CYP3A5, and to a lesser extent also for CYP2C9 [23,27,28]. CYP2D6 metabolizes about 30% of all drugs on the market and is thus the second most important CYP in drug metabolism after CYP3A4 [29]. 

### 2.4. iPSC-Derived Hepatocytes

Induced pluripotent stem cells (iPSCs) can be generated from any human cell type and then be differentiated into hepatocytes. This allows researchers to obtain material from patients or healthy donors without the need of a liver biopsy. Cellular material can be obtained from individuals with particular phenotypes or genotypes that might be of special interest to study DILI [30,31]. So far, many attempts have been made to generate functional adult human hepatocytes from iPSCs, but the data indicate that hepatocytes derived from iPSCs (iPSC-heps) show a more fetal-like state instead of displaying mature adult liver cell functions [32,33,34,35,36,37]. However, iPSC-derived hepatocytes are capable of albumin synthesis, glycogen storage and CYP expression and they resemble adult primary hepatocytes more closely than different hepatoma cell lines including HepG2 [38]. A recent study reported a more mature phenotype of iPSC-derived hepatocytes. In this study, albumin synthesis capacity and expression of several drug-metabolizing phase I and phase II enzymes reached similar levels than pHHs [39]. Further research on differentiation and culture conditions might lead to a stable mature phenotype of those cells, but as of yet, iPSC-heps should not be the first choice for toxicological analyses.

### 2.5. Upcyte Hepatocytes/Primary-Like Hepatocytes

The upcyte technology uses lentiviral transfer of proliferation inducing genes into primary human hepatocytes. This extends the lifespan of pHHs to up-to 30 cell-doubling times upon the maintenance of major primary characteristics [40,41,42,43]. Development of the technique, the so called second-generation upcyte technology, led to further improvement in those hepatocytes regarding the maintenance of polarity, expression of drug-metabolizing enzymes as well as their suitability for drug interaction studies [44,45]. Therefore, these primary-like cells combine the advantages of primary liver cells with the ease of use and extended availability of cell lines. These cells represent a proliferating liver cell system that can be propagated in vitro to achieve high cell numbers for large experimental setups or repetition of experiments with cells from the same donor. However, the cells are contact-inhibited and do not further proliferate when seeded at high-density; therefore, they resemble quiescent cells, such as it is the case in the liver in vivo. A direct comparison of gene expression profiles showed that upcyte hepatocytes more closely resembled pHHs than HepG2 cells [46]. Capability of albumin synthesis, urea secretion and glycogen storage were similar to pHHs, while activity of CYP enzymes strongly depended on donor characteristics. CYP1A2 and CYP2C19 activity could not be detected in any of three donors studied and activities of CYP2C9, CYP2D6 and UGT2B7 were lower in upcyte hepatocytes than in pHHs. However, CYP2B6, CYP3A4 and UGT1A1 enzyme activities were comparable or even higher in upcyte cells [46]. 

Others have described an alternative method to keep pHHs in culture for an extended time period. One group describes a protocol to convert pHHs into liver progenitor-like cells that can be expanded for many passages in vitro. The expanded cells can be converted back into mature hepatocytes at any desired time-point using specialized hepatocyte maturation media [47]. Others keep pHHs proliferating by using a complex medium composition and hypoxic conditions [48].

As the iPSC technology, those methods create the possibility of using material from different (healthy) donors, but with the advantage of generating cells with a mature liver phenotype.

### 2.6. Summary of Human Liver In Vitro Models

Obviously, pHHs are the most relevant model to study the human liver in vitro. In recent years, HepaRG cells have become a widely used substitute for pHHs with properties that closely resemble pHHs. Upcyte and other primary-like hepatocytes might become of interest for toxicological analyses since they also display mature hepatic features and have the advantage that they can be generated from different donors. HepG2 cells are widely used, but as hepatoblastoma cell line, they lack many hepatic properties. Hepatocytes differentiated from iPSCs still show only fetal-like hepatic characteristics and functions but might become more valuable for hepatotoxic assay if the maturation protocols are optimized. Table 1 summarizes the main features of the described hepatic cell types.

## 3. Three-Dimensional Culture Models for Human Hepatocytes

All the above-mentioned cell types have been used in different types of 3D culture. 3D cultivation more closely mimics the human liver and, indeed, many hepatic functions and properties could be restored or improved upon 3D culture. The reason for this seems to be improved cell-cell- as well as cell-matrix-communication. Cell junctions between hepatocytes improve their functionality and it has been shown, for example, that albumin secretion and importantly also CYP expression rely on adherence junctions. Further, hepatic homeostasis is also maintained by cell-extracellular matrix interactions via integrin signaling pathways (reviewed in [49]). The following sections will discuss well-established and emerging 3D culture systems for human hepatocytes. Efforts in the field are being made to generate stable and reliable test systems for toxicology analyses of liver cells with the aim of creating an environment for the cells that allows researchers to mimic the human liver in vitro. 

### 3.1. Liver Spheroid and Organoid Culture

Spheroids are formed by self-aggregation of hepatocytes when cultured in suspension without any substrates that promote cell attachment, such as collagen I. This can be achieved by the hanging drops method or by using ultra-low attachment (ULA) multiwell plates. As the starting cell number can be defined, those spheroids usually have equal sizes, which is a feature of importance for standardized measurements. Further, they can be kept in culture for several days to weeks. Spheroids can also be formed in stirred tank bioreactors. This method allows us to generate spheroids on a large scale, but sizes may vary greatly, which makes it difficult to compare and to reproduce data. Further, single spheroids must be isolated from the stirred tank bioreactor to perform individual experiments as spheroids are generated in a single reaction tank. Due to these reasons, the hanging drops or ULA method are the preferred ones for toxicological analyses and, if not otherwise stated, results described below refer to spheroids generated by those two methods. 

Recent advances were also made for liver organoid cultures. Such organoids derive from adult liver cells or iPSCs, are more difficult to create and need a scaffold of extracellular matrix (often supplemented with growth factors and cytokines) for aggregation. In contrast to conventional spheroids, mature organoids consist of different cell types including also non-parenchymal cells (NPCs) or biliary epithelial cells.

#### 3.1.1. Spheroids from Primary Human Hepatocytes

Many attempts were made to use pHHs in 3D culture with the aim to maintain and to prolong their viability and to prevent dedifferentiation. PHH spheroids were viable for at least 2 and up to 7 weeks with stable albumin production [50,51,52,53,54], urea synthesis [51,52] and glycogen storage [54]. Additionally, cellular polarization was clearly present in pHH spheroids as shown by MRP2 [51,53], P-glycoprotein (Pgp), [54], and BSEP expression [53,54]. Drug-metabolizing enzyme expression and activity of CYP1A2 [53,55,56,57], CYP2B6 [55], CYP2C9 [55], CYP2C19 [58], CYP2D6 [53,55,57], CYP3A4 [50,53,55,56,57,58,59], and UGTs [56,59] was stable over several weeks of pHH spheroid culture. CYP2C9 activity even increased with culture time [53,55,56,58], while CYP2C8 [53,56], and in some cases also CYP1A2, CYP2B6, CYP2D6 and CYP3A4 [55,58] activities decreased with time. In general, maintenance of their initial hepatic transcriptomic and metabolomic profiles was possible for at least 2–5 weeks [51,53,59,60], which allowed long-term and repeated-dose toxicity testing. EC50 values for acetaminophen, bosentan, diclofenac, troglitazone, and other hepatotoxic compounds significantly decreased upon long-term treatment [53,60] and were closer to the corresponding in vivo C_max_ than acute toxicity EC50 values [53,59]. Importantly, liver spheroids cultured in chemically-defined medium reached 100% specificity and 69% sensitivity when 123 hepatotoxic compounds were tested. The model system could reliably distinguish between hepatotoxic substances and their nontoxic structural analogues [61]. The authors used a repeated-dose approach for 14 days and an easy-to-handle ATP assay as readout. Such comprehensive studies corroborate the utility of pHH spheroids in drug development, toxicity testing and DILI prediction. Further, those studies used panels of drugs with different toxicity mechanisms, including mitochondrial dysfunction, steatosis, cholestasis, and fibrosis. This implies that spheroid cultures of pHHs can be used to study different mechanisms of drug induced hepatotoxicity [53,59,60,61,62,63].

Spheroids can also be formed by using pHHs together with non-parenchymal cells (NPCs). This approach intends to further improve hepatic characteristics. Those spheroids, often called microtissues, displayed pronounced glycogen storage and albumin synthesis capabilities as well as expression of CYP1A2, CYP2C9 and CYP3A4. Maintenance of cell polarity could be shown by MRP2 and BSEP expression [64,65,66]. Viability of the spheroids could be maintained for up to 7 weeks in one of the studies, but albumin secretion declined from day 28 on. Interestingly, *CYP1A1/2*, *CYP2C9* and *CYP3A4/5* transcript levels were higher in those microtissues than in clinical liver specimens or pHHs upon long-term culture [65,66]. However, on protein level, only CYP2B6 and UGT1A1 levels remained stable for 7 weeks of culture. Other CYP and phase II enzyme protein levels dropped already at day 7 of culture. Additionally, a decline of CYP1A2, CYP2B6, CYP2C9 and CYP2C19 activities could be observed after 4 weeks in culture, which is in accordance with the reduction in albumin production at this time-point [66]. Others directly compared hepatic spheroids in monoculture and in co-culture with NPCs over a culture time of 14 days. Spheroid functionality was increased in the co-culture setup as shown by expression of albumin (*ALB*) and *CYP3A4*, as well as by albumin synthesis [67]. A contradictory study, however, showed that *ALB* and *CYP1A2* and *CYP3A4* expression was higher in hepatocyte-only spheroids than in NPC-containing spheroids [68]. Thus, it remains to be shown whether co-culture with NPCs really adds a benefit to pHH spheroid culture.

Other studies used such 3D human liver microtissues for comprehensive toxicology analyses and tested a panel of 100 and 110 hepatotoxic compounds, respectively. Results were directly compared with 2D pHH cultures. It must be noted that spheroids were treated for 7 and 14 days, while monolayer cultures were treated for 24 and 72 h, respectively. Overall, specificity of spheroids for detection of hepatotoxic substances was high (79–85%), and sensitivity was moderate (19–61%) indicating that also NPC containing spheroid cultures of pHHs are suitable for hepatotoxicity risk assessment in drug development processes and for the study of toxicity mechanisms [69,70]. 

Further improvement of spheroid co-cultures might be achieved by using bioprinting techniques. Here, spheroids are formed by bioprinting pHHs together with non-parenchymal cells such as endothelial and stellate cells on transwell culture inserts of multi-well plates [71]. Upon this culture system, albumin synthesis and glycogen storage capacity could be sustained, and albumin secretion even increased over the culture period of 4 weeks. Expression levels of *CYP1A2*, *CYP2B6*, *CYP2C9*, *CYP2D6* and *CYP3A4* also increased significantly over time, with a peak at day 14 followed by a slight decrease at day 28. In addition, EC50 values for trovafloxacin were lower in those so-called 3D liver tissues than in corresponding 2D pHH cultures [71]. Bioprinting methods are emerging technologies, are often expensive and difficult in handling. Further studies are required to provide easy-to-use and reproducible protocols and to prove the utility of the method for toxicology analyses.

Primary liver characteristics could also be maintained when spheroids were generated in a stirred tank bioreactor. The authors described stable urea and albumin synthesis as well as stable expression of phase I enzymes (*CYP1A2*, *CYP2C9*, *CYP3A4*), the phase II enzymes *GSTA1* and *UGT2B7* and the polarization marker F-actin [72]. Gene expression of *CYP1A2*, *CYP2C9* and *CYP3A4* could be further improved in stirred tank bioreactors by adding an outer layer of bone marrow-derived mesenchymal stem cells to the liver spheroids, while albumin and urea production remained unaffected with this strategy [73]. The greatest disadvantage of spheroids in stirred tank bioreactors is the single reaction vessel. Spheroids generated by this method a) vary greatly in size and b) need to be separated and transferred to other culture vessels for toxicology testing. Therefore, this method is not suitable for high-throughput analyses and spheroids generated by the hanging drops or ULA method should be preferred.

#### 3.1.2. Spheroids from HepG2 Cells

Many groups use hepatoma cell lines as HepG2 for spheroid culture due to their ease of use and low cost. It could be shown that culture in this 3D format indeed improves hepatic properties at several levels.

Albumin synthesis [74,75,76,77,78] and urea production [76] were elevated when HepG2 cells were cultured as spheroids and compared to their 2D cultured counterparts. However, one study showed a contradictory result: albumin secretion levels were significantly higher in 2D than in 3D spheroid cultures [79]. Gene expression of drug-metabolizing enzymes such as *CYP1A1/2* [74,75,78,80], *CYP3A4* [74,75,80] and *UGT1A1* [74,75] could be increased upon 3D spheroid culture. Importantly, in addition, cellular polarization could be restored. This was shown by positive MRP2 and Pgp staining as well as by functional transporter assays indicating presence of canalicular-like structures [76].

Co-culture of HepG2 cells with endothelial cells and application of a cell coating technique resulted in elevated albumin synthesis, MRP2 expression and CYP enzyme activities [81]. Similarly, HepG2 cells cultured together with endothelial and mesenchymal cells as tubular 3D liver microtissues had higher expression levels of drug-metabolizing enzymes than 2D cultured counterparts [82].

Additionally, bioprinted 3D HepG2 spheroids showed stable albumin and urea synthesis for at least 7 days [83,84]. Such bioprinted HepG2 spheroids displayed higher *CYP1A2* gene expression and higher sensitivity towards acetaminophen than 2D cultured HepG2 cells [84]. Bioprinting HepG2 cells together with fibroblasts resulted in elevated albumin production [85]. 

Cell lines as HepG2 are by nature very well suited for high-throughput screens, which is desirable for any toxicological test system. Indeed, HepG2 cells cultured in 3D spheroids were more susceptible to hepatotoxic compounds and showed considerably lower EC50 values for many different drugs than 2D cultured HepG2 cells [76]. However, other studies have shown that HepG2 spheroids were more resistant to hepatotoxins than their 2D counterparts, both in acute toxicity and repeated-dose toxicity experiments [86]. Furthermore, they were still considerably less sensitive to hepatotoxic compounds than 3D cultured pHHs. This might be explained by the low expression of CYP and other drug-metabolizing enzymes. As their basal expression is very low to absent in HepG2 cells, also an increase in expression levels upon spheroid culture might not lead to sufficiently elevated enzyme activities. Therefore, the value of such cells for drug development and DILI prediction is questionable.

#### 3.1.3. Spheroids from HepaRG Cells

HepaRG cells also benefit from spheroid culture conditions. They displayed improved albumin synthesis capacity upon culture as spheroids in hanging droplets and when compared to HepaRG monolayer culture. Those HepaRG spheroids needed several days for maturation. Albumin synthesis was elevated at day 6 of spheroid culture, and mRNA levels of *CYP1A2*, *CYP2B6* and *CYP3A4* first dropped, then slightly increased at day 4 and were elevated 1.2–3 -fold at day 7 when compared to day 0 [77]. Additionally, others have shown that HepaRG spheroids (self-aggregated or bioprinted) could be cultured over several weeks and that those cultures maintained several hepatic properties: HepaRG spheroids were capable of (a) albumin [87,88,89,90,91] and urea [87,90] production, (b) displayed expression and activity of CYP1A2 [88,89], CYP2B6 [89] and CYP3A4 [86,88,89,90,92], (c) showed phase II enzyme activity [89,91], and (d) displayed cellular polarization shown by MRP2 [87,88,90] and Pgp [91] expression, and by F-actin bands, indicative of bile canalicular structures [89,91,92]. Regarding expression and activity of phase I enzymes, however, several studies have shown that basal levels in spheroids were unaffected, in part even slightly lower than in monolayer cultures. This might be explained by the relatively high basal expression of several CYP enzymes in HepaRG cells. However, the authors of those studies have deemed it necessary to elevate expression and activity of CYPs by induction using β-naphthoflavone (for CYP1A2), phenobarbital (for CYP2B6) or rifampicin (for CYP2C9 and CYP3A4) to reach more relevant levels [87,89,91,93].

Importantly, and in accordance with the presence of hepatic characteristics, HepaRG spheroids were also more sensitive to hepatotoxic compounds than monolayer cultures. This was shown by smaller EC50 values in a range of substances including acetaminophen, tamoxifen, and aflatoxin B1 [86,88,90,91,94]. Interestingly, a recent study showed in a high-throughput 384-well format that HepG2 spheroids were more sensitive to hepatotoxic compounds than HepaRG spheroids. When 150 compounds were tested, HepG2 spheroids displayed a sensitivity of 58% and a specificity of 83%, while HepaRG spheroids showed sensitivity of 47% and specificity of 86% [95]. Static HepaRG spheroid cultures could also be combined with lactate and oxygen microsensors. This allowed live, long-term, and fast measurement of the cellular metabolic activity. This test system was validated using antimycin A and bosentan as test substances, and lactate production increased or decreased accordingly [96]. Further, it was shown that HepaRG spheroids were able to predict steatosis and mitochondrial dysfunction, while cholestasis could only be predicted in 2D culture models in this work [88]. Another study presented spheroid models to study cholestatic liability of compounds and showed that HepaRG spheroids as well as pHH spheroids were both able to reliably predict cholestasis [62]. A model to detect liver fibrosis could be generated by co-culturing HepaRG cells and hepatic stellate cells in spheroid cultures [97]. Those studies imply that HepaRG cells are well suited to study different types of hepatotoxicity.

Culture of HepaRG as 3D spheroids indeed seems a promising system to maintain primary hepatic properties. However, some studies found that expression of drug-metabolizing enzymes needed to be elevated by prototypic CYP inducers. This, together with the required time-consuming differentiation procedure, renders the system somewhat complicated for toxicity testing. In summary, however, HepaRG cells constitute a more reliable test system than HepG2 cells, especially regarding their drug-metabolizing enzyme expression, and more closely resemble pHHs than other cell lines of cancerous origin.

#### 3.1.4. Spheroids from iPSC-Derived Hepatocytes

Studies on spheroid cultures of iPSC-heps for toxicity testing are still rare. It was shown that hepatic properties improved upon 3D culture of those liver cells, but they still maintained a more fetal-like phenotype, and adult marker genes (including *CYP1A2*, *CYP2C9*, *CYP3A4* and *ALB*) were only expressed at low levels [98,99,100]. For example, albumin production was not significantly increased when iPSC-heps were cultured as spheroids instead of 2D monolayers. However, an increase in urea production could be observed at least at day 12 of spheroid culture [99]. In contrast, others have shown that spheroids of iPSC-heps were capable of albumin production, urea synthesis, glycogen storage and gene expression of drug-metabolizing enzymes (*CYP1A2, CYP2C9, CYP2C19, CYP2D6, CYP3A4, UGT2B7*). However, again, levels remained far below those of pHHs [101]. A very recent study compared 2D and 3D cultured iPSC-heps with pHHs cultured in 2D and 3D spheroids. Basal activities of CYP1A2, CYP2B6 and CYP3A4, as well as canaliculi formation, were similar in all culture systems, but CYPs could be induced successfully only in pHH cultures, which is an important feature of functional human hepatocytes [102].

Bioprinted 3D spheroids of iPSC-heps displayed higher albumin and urea synthesis as well as *CYP1A2* and *CYP3A4* expression than their 2D cultured counterparts [103]. Co-culturing of iPSC-hep spheroids with endothelial cells seems to improve maturity of the cells as shown by albumin and urea secretion and CYP enzyme activities [104].

Interestingly, iPSC-hep spheroids were more sensitive to a set of 24 hepatotoxic compounds than HepG2 spheroids in one study [100], while another study showed opposite results for 10 out of 23 tested compounds [105]. When compared to pHH spheroids, IC50 values of iPSC-hep spheroids were similar in 12/15 tested hepatotoxic compounds [101]. In another study, the authors tested a set of seven hepatotoxic compounds and found that pHHs generally displayed higher sensitivity [102].

Hepatic differentiation of iPSCs is a promising approach for modelling the human liver in vivo. To date, however, those hepatocytes have a more fetal-like than mature phenotype and efforts must be made to optimize the differentiation procedure towards adult characteristics. Three-dimensional cultures seem to add only little towards this goal, and contradictory data on utility for toxicological analyses render this system, still, an immature technology for such applications.

#### 3.1.5. Spheroids from Upcyte and Primary-Like Hepatocytes

Data for spheroid culture of upcyte or other primary-like hepatocytes are even scarcer than those for iPSC-heps. Despite their characteristics seem to make them a promising tool for in vitro toxicology testing, they might be still rather unknown in the community. One study compared gene expression of drug-metabolizing enzymes in 2D and 3D spheroid cultures of upcyte hepatocytes. The results indicate that expression levels of *CYP2C19, CYP3A4, MRP2* and *OATP-C* in spheroids were slightly higher than in 2D cultures, while *CYP2C8* and *BSEP* expression was reduced in 3D cultures [41]. A second study used spheroids of primary-like hepatocytes generated from proliferation-competent liver progenitor cells. Those spheroids showed elevated *ALB* and *CYP1A2, CYP2D6, CYP3A4, UGT1A1* and *MRP2* expression, albumin and urea synthesis as well as glycogen storage. However, these levels did not reach those of pHHs. Gene-expression profiling showed that spheroids of primary-like hepatocytes clustered with pHHs and differentiated HepaRG cells, but not with HepG2 cells, indeed indicating a mature phenotype [106]. And a third study showed that spheroids of so called ProliHHs were comparable to pHHs regarding albumin expression and secretion, but expression and activity levels of CYP1A2, CYP2B6 and CYP3A4, as well as *BSEP* and *MRP2* expression levels were considerably lower than in pHHs [48].

Future works need to characterize spheroids of such primary-like cells thoroughly, and especially show their utility for toxicology analyses.

#### 3.1.6. Organoids from Human Primary and iPSC-Derived Hepatocytes

It was shown that organoids from adult liver cells were capable of albumin secretion, glycogen storage, and CYP expression and activity. Their gene expression profiles were highly similar to those of pHHs. Further, organoids from adult liver cells can be cultured for up to 2.5 months [107,108,109]. Organoid cultures of iPSC-heps also resulted in improved hepatic properties with stable albumin, CYP3A4 and BSEP expression, but levels were considerably higher in pHHs. However, a functional bile canaliculi network could be successfully established [110,111]. Co-culture of organoids from iPSC-heps with endothelial cells seems to improve hepatic functions [112].

Liver organoids are an emerging technology and, as yet, no data are available on their suitability for toxicological testing.

### 3.2. Perfused Bioreactors and Liver-on-a-Chip Models

In this section, 3D culture systems of different hepatic cell systems in perfused bioreactors will be discussed. Perfused bioreactors comprise hollow-fiber bioreactors, but also more sophisticated microfluidic bioreactors, often referred to as organ- (or liver-) on-a-chip models.

Hollow-fiber bioreactors consist of tubular modules where cells are immobilized. Those tubules contain capillaries that are perfused with medium and gases, mimicking capillary blood flow. However, such bioreactors require high volumes of culture medium (around 800 mL) as well as a high starting number of cells (around 10^10^ cells or more) for seeding [113,114]. These features are not compatible with high-throughput screening for toxicological analyses. Small scale perfused bioreactors also require much higher cell numbers than assays in multi-well formats. However, hollow-fiber bioreactors are a valuable tool for the long-term culture of hepatocytes with maintenance of their primary characteristics and functions.

Organ-on-a-chip systems for liver cells are miniaturized perfused bioreactors that usually consist of liver cells embedded in an extracellular matrix and sometimes are also co-cultured with Kupffer, stellate and/or endothelial cells. Culture medium perfusion not only mimics blood flow, but also shear stress and a gradient of oxygen and nutrient supply as it can be observed along the lobular axis in the human liver [115]. Some of those liver-on-a-chip systems even provide the possibility of automated microscopy for live cell imaging [116,117,118,119]. Such technical advances pave the way for high-throughput screens with those devices, despite they are still difficult to handle and expensive.

#### 3.2.1. Perfused Bioreactors with Primary Human Hepatocytes

As with spheroid cultures, embedding cells in the 3D environment of a perfused bioreactor has beneficial effects on primary hepatic properties. PHHs could be cultured in hollow-fiber bioreactors for up to 3 weeks and urea and albumin production remained stable from day 5–7 onward, after an initial drop of about 50% [113]. Attempts of downscaling the hollow-fiber bioreactor format were made to make the technique more suitable for studying hepatotoxic effects of newly developed compounds. This resulted in a culture medium reduction to 8 mL or 2 mL without further downregulation of albumin [120,121] and urea synthesis, and also MRP2 and Pgp expression were sustained [121]. Unfortunately, expression and activity of several drug-metabolizing CYP enzymes decreased over the time of culture [120]. Other studies on miniaturized hollow-fiber bioreactors showed that albumin and urea production as well as CYP1A2 and CYP3A4 activities were stable for at least 10 days. Activities of CYP2B6, CYP2C9 and CYP2D6 decreased over the time course [122]. In summary, data do not indicate that pHHs should be cultured in hollow-fiber bioreactors for toxicological analyses.

A further step towards miniaturization was achieved by the development of several human liver microphysiology systems. Those liver-on-a-chip models have the great advantage of mimicking liver zonation and thus creating the possibility to better recapitulate the physiology and function of the human liver in vitro. Oxygen concentration gradients ranged from 3–13% in such models and cells close to the perfusion (recapitulating the oxygen-rich periportal zone) displayed higher levels of albumin and urea synthesis than cells in the oxygen-poor region (recapitulating the central vein region) [115]. In contrast, CYP expression and acetaminophen toxicity was higher in the oxygen-poor region [115]. CYP2D6, CYP2C19 and CYP3A4 activities were stable for at least 10 days of culture on chips, while CYP1A2 activity slightly decreased over this time period. Interestingly, no difference could be observed between liver spheroid and liver-chip cultures when sensitivity to acetaminophen and fialuridine as well as CYP activity profiles were directly compared [58].

Upon co-culture with NPCs under microfluidic conditions, pHHs retained albumin [123,124] and urea [124] secretion and polarization as shown by BSEP expression and MRP2 activity [123,125]. *CYP1A2*, *MRP1* and *UGT1A5* levels were similar to those of freshly isolated pHHs [125]. Interestingly, shear stress seems to have a positive impact on hepatic functions as albumin secretion and CYP1A2 and CYP2D6 activities were higher than under static conditions [124]. Further, the co-culture system could recapitulate toxicities of several hepatotoxic compounds [123]. 

Even if hepatic properties more closely reflect the in vivo situation when microfluidic systems are used, so far, they seem not to perform better in toxicological analyses than spheroid cultures of pHHs. Future studies in the field will show whether such emerging technologies are of use in drug development processes and DILI prediction studies.

#### 3.2.2. Perfused Bioreactors with HepG2 Cells

Interesting approaches combine different 3D culture systems of HepG2 cells. Often, HepG2 spheroids, in the first instance, are generated to improve their hepatic characteristics. Those spheroids are then used either for bioprinting or for direct culture in liver-on-a-chip devices under microfluidic conditions. For example, HepG2 spheroids were embedded in a hydrogel scaffold and then bioprinted on a liver-on-a-chip device. Those spheroids further grew for 30 days under microfluidic conditions, maintained biomarker expression and, importantly, recapitulated toxicity of acetaminophen [126]. Further, HepG2 spheroids were cultured in a microfluidic device mimicking shear stress and an oxygen gradient similar to the in vivo situation. Those spheroids showed high levels of albumin and urea synthesis as well as MRP2 expression. Additionally, expression levels of most drug-metabolizing, phase I and phase II enzymes were at least 20-fold higher than in 2D cultured HepG2 cells [127]. Others directly compared functions of conventional HepG2 spheroids with HepG2 spheroids cultured in a microfluidic device. The latter showed higher albumin and urea synthesis capacities as well as higher Pgp expression [128]. More specialized microfluidic bioreactors are designed for real-time monitoring of oxygen consumption and lactate production. In such devices, HepG2 cells were embedded in a collagen I matrix together with oxygen sensor beads. Cells could be cultured for up to 30 days and proved to be suitable for toxicological analysis as shown by testing hepatotoxic compounds, including acetaminophen, amiodarone and troglitazone [116,118,119]. 

Co-culture of HepG2 spheroids with non-parenchymal cells on microfluidic liver-on-a-chip devices resulted in cell polarization as shown by MRP2 expression. Albumin and urea synthesis were significantly higher under shear stress than under static conditions, and CYP1A2 and CYP3A4 activities were increased [129]. Bioprinting was used to create structured spheroids consisting of HepG2 and endothelial cells to mimic vascularization. The structured and perfused spheroids displayed stable urea synthesis over 10 days, and albumin synthesis increased over this culture time [130].

It must be noted that these studies mainly focused on the development of such 3D culture systems and only a few of them used hepatotoxic compounds to test the utility of those models for toxicological analyses. HepG2 cells might be well suited for the establishment of such systems, but due to their low drug-metabolizing enzyme expression even in 3D cultures, other cell models should be preferred for compound development and DILI prediction.

#### 3.2.3. Perfused Bioreactors with HepaRG Cells

There are few studies on HepaRG cells in perfused bioreactors. It was shown that the expression of the basolateral uptake transporter OATP1B1 was significantly lower in bioreactor cultured HepaRG cells than in pHHs under same conditions. In contrast, CYP3A4 expression was comparable between HepaRG and pHHs in perfused bioreactor cultures [131]. Culture of HepaRG cells in microfluidic plates resulted in good viability and stable polarization patterns for 14 days [132,133] as well as stable albumin production and response to acetaminophen treatment [132].

Upon co-culture with macrophages, endothelial and stellate cells, CYP3A4 protein expression and albumin and urea secretion were significantly increased, MRP2 expression could be maintained, and cellular viability upon treatment with hepatotoxic compounds was observed in real time by measuring oxygen consumption [134].

As HepaRG cells are a well-studied tool for toxicological analyses, it can be expected that with the further development of perfused bioreactors, more data using HepaRG in such systems will be generated.

#### 3.2.4. Perfused Bioreactors with iPSC-Derived and Upcyte Hepatocytes

Similar to HepaRG, only a few studies used iPSC-heps in perfused bioreactors. In a hollow-fiber bioreactor, iPSC-heps produced more albumin than 3D spheroid or 2D cultured counterparts, but urea production was even lower than in 2D culture. CYP1A2 and CYP3A4 activities were elevated, but still significantly lower than in pHHs [99].

An interesting approach showed that it is possible to perform the iPSC differentiation procedure directly on a microfluidic chip system. Starting from day 20 of the differentiation procedure, albumin and CYP3A4 mRNA and protein expression levels were increasing. Of note, those levels were even higher in perfused than in static conditions. Glycogen storage, albumin and urea synthesis were stable for up to 7 weeks of culture, and the model was responsive to acetaminophen treatment [135]. Another approach consisted of the co-culture of iPSC-heps with endothelial cells and macrophages on a microfluidic liver-on-a-chip model. Albumin and urea synthesis were stable over a culture time of 2 weeks, and CYP3A4 activity levels increased over time. Further, 159 compounds were tested on this system showing that it is suitable for high-throughput screens, but sensitivities and specificities were unfortunately not calculated [136]. To date, there are no published data on upcyte or other primary-like hepatocytes in perfused bioreactors.

In summary, both the cell models and the 3D culture techniques described here need a lot of improvement. Future studies need to show if and how such models can be of value for toxicological testing.

## 4. Conclusions and Outlook

Most hepatotoxicity studies on liver 3D models use spheroids of primary human hepatocytes and data suggest that this system can accurately and stably predict DILI for a large number of compounds. Consequently, this culture model can be seen as the gold standard when it comes to recapitulation of human liver characteristics in vitro with the aim of performing hepatotoxicity studies. Using material from patients or donors also allows researchers to study distinct genotypes or phenotypes. This is of great importance when distinct hepatic phenotypes are required to study different toxicity mechanisms and should be considered when choosing an appropriate model for specific research questions. Most likely, the largest advantage of 3D culture of pHHs is the long-term maintenance of primary characteristics over several weeks; thus, allowing not only acute toxicity testing but also long-term and repeated-dose hepatotoxicity studies. Phase I and phase II enzyme expression and activity as well as cellular polarization can be maintained over several weeks of 3D culture. The culture model also allows for the study of different mechanisms of hepatotoxicity.

The utility of HepG2 cells for such purposes remains questionable as hepatic functions remain at a low basal level, also apparent in 3D culture. In contrast, HepaRG cells benefit from 3D cultures by maintaining or improving their primary characteristics. Data for iPSC-heps and upcyte/primary-like cells are still scarce and more research is needed to get a clearer picture on how suitable those models are for toxicological studies.

The data presented here allow for the conclusion that both pHHs and HepaRG spheroids are suitable for predicting hepatotoxicity and also allow for the determination of different toxicity mechanisms such as fibrosis, mitochondrial dysfunction, cholestasis or steatosis. It should be noted that co-culture of hepatocytes with non-parenchymal cells might have additional benefits.

Among the perfused bioreactor systems, large-scale hollow-fiber reactors can be neglected for toxicity testing due to their large format. Miniaturized microfluidic liver-on-a-chip systems are an emerging technology with many advantages including the possibility of mimicking liver zonation and co-culture with NPCs, but they are often difficult to handle, expensive, and not always suited for high-throughput screens. Further, comprehensive studies on their suitability for predicting DILI are still lacking. 

Similar to liver-on-a-chip models, bioprinting methods also seem to be promising emerging technologies. Importantly, bioprinting can both be used for creating liver-like tissues in static models similar to spheroid cultures, as well as for creating 3D liver tissues on perfused bioreactors. However, data on bioprinted liver tissues are still scare and future studies need to show their suitability for toxicological analyses, also in high throughput.

Table 2 gives an overview of the characteristics of different 3D culture systems for toxicity testing using distinct cell types.

Liver spheroid cultures of pHHs and HepaRG cells offer a reliable, robust, and reproducible tool for studying hepatotoxicity and predicting DILI in vitro. Alternative cell types such as iPSC-heps or upcyte hepatocytes might gain relevance in the future. In addition, emerging technologies in relation to 3D liver in vitro systems are promising and further developments and breakthroughs in the field can be expected. In-depth characterization and comprehensive benchmarking of any novel 3D liver culture method for toxicological testing is urgently needed to gain relevance at an early stage of drug development.

## Figures and Tables

**Table 1:** Main characteristics of human in vitro hepatocyte models in conventional 2D culture: a qualitative assessment.

	pHHs	HepG2	HepaRG	iPSC-heps	Upcytes
Availability	+	+++	+++	++	++
Low costs	-	+++	+	-	+
Ease of use	++	+++	+ ^1^	+ ^1^	++
Proliferation/lifespan	-	+++	+++	+++	++ ^2^
Albumin synthesis	+++	+	++	+	+++
Urea production	+++	+	-	+	+++
Glycogen storage	+++	+++	+++	+++	+++
Phase I enzyme expression	+++	-	++ ^3^	+ ^4^	++
Phase II enzyme expression	+++	-	+++	+ ^4^	++

^1^ Differentiation procedure required; ^2^ Limited lifespan, not immortalized; ^3^ Poor metabolizer for CYP2D6; ^4^ More fetal-like than adult expression patterns. pHHs: primary human hepatocytes; iPSC-heps: induced pluripotent stem-cell-derived hepatocytes; upcytes: upcyte or primary-like hepatocytes. Qualitative assessment: (-): absent; (+): low/poor; (++): intermediate/moderate; (+++): high/good.

**Table 2:** Main characteristics of hepatic 3D culture techniques for in vitro toxicological analyses.

	Ease of Use	Long-Term Maintenance of Hepatic Properties	Suitability for DILI Prediction	Compatible with High Throughput Screening
**Spheroids**				
pHHs	++	+++	+++	++
HepG2	+++	+	+	+++
HepaRG	+ ^1^	++	+++	+++
iPSC-heps	+ ^1^	+ ^2^	+	++
upcytes	++	n.d.	n.d.	++
**Perfused Bioreactors**				
pHHs	+	+++	+++	+
HepG2	++	+	n.d.	++
HepaRG	+ ^1^	++	n.d.	++
iPSC-heps	+ ^1^	+ ^2^	n.d.	+
upcytes	+ ^3^	n.d.	n.d.	+ ^3^

^1^ Differentiation procedure required; ^2^ More fetal-like than adult expression patterns; ^3^ no data available, hypothesized assessment. pHHs: primary human hepatocytes; iPSC-heps: induced pluripotent stem-cell-derived hepatocytes; upcytes: upcyte or primary-like hepatocytes. Qualitative assessment: (+): poor; (++): moderate; (+++): good; n.d.: not enough data available.

## Data Availability

Not applicable.

## References

[1] Kiamehr M., Heiskanen L., Laufer T., Düsterloh A., Kahraman M., Käkelä R., Laaksonen R., Aalto-Setälä K. (2019). Dedifferentiation of Primary Hepatocytes is Accompanied with Reorganization of Lipid Metabolism Indicated by Altered Molecular Lipid and miRNA Profiles. Int. J. Mol. Sci..

[2] Lauschke V.M., Vorrink S.U., Moro S.M.L., Rezayee F., Nordling Å., Hendriks D.F.G., Bell C.C., Sison-Young R., Park B.K., Goldring C.E. (2016). Massive rearrangements of cellular MicroRNA signatures are key drivers of hepatocyte dedifferentiation. Hepatology.

[3] Fisher K., Vuppalanchi R., Saxena R. (2015). Drug-induced liver injury. Arch. Pathol. Lab. Med..

[4] Lasser K.E., Allen P.D., Woolhandler S.J., Himmelstein D.U., Wolfe S.M., Bor D.H. (2002). Timing of new black box warnings and withdrawals for prescription medications. JAMA.

[5] Russo M.W., Galanko J.A., Shrestha R., Fried M.W., Watkins P. (2004). Liver transplantation for acute liver failure from drug induced liver injury in the United States. Liver Transpl..

[6] Ostapowicz G., Fontana R.J., Schiødt F.V., Larson A., Davern T.J., Han S.H., McCashland T.M., Shakil A.O., Hay J.E., Hynan L. (2002). Results of a prospective study of acute liver failure at 17 tertiary care centers in the United States. Ann. Intern. Med..

[7] Zhou Y., Shen J.X., Lauschke V.M. (2019). Comprehensive Evaluation of Organotypic and Microphysiological Liver Models for Prediction of Drug-Induced Liver Injury. Front. Pharmacol..

[8] Chapman K.L., Holzgrefe H., Black L.E., Brown M., Chellman G., Copeman C., Couch J., Creton S., Gehen S., Hoberman A. (2013). Pharmaceutical toxicology: Designing studies to reduce animal use, while maximizing human translation. Regul. Toxicol. Pharmacol..

[9] Aleksunes L.M., Campion S.N., Goedken M.J., Manautou J.E. (2008). Acquired resistance to acetaminophen hepatotoxicity is associated with induction of multidrug resistance-associated protein 4 (Mrp4) in proliferating hepatocytes. Toxicol. Sci..

[10] Kozyra M., Ingelman-Sundberg M., Lauschke V.M. (2017). Rare genetic variants in cellular transporters, metabolic enzymes, and nuclear receptors can be important determinants of interindividual differences in drug response. Genet. Med..

[11] Zhou Y., Ingelman-Sundberg M., Lauschke V.M. (2017). Worldwide Distribution of Cytochrome P450 Alleles: A Meta-analysis of Population-scale Sequencing Projects. Clin. Pharmacol. Ther..

[12] López-Terrada D., Cheung S.W., Finegold M.J., Knowles B.B. (2009). Hep G2 is a hepatoblastoma-derived cell line. Hum. Pathol..

[13] Aden D.P., Fogel A., Plotkin S., Damjanov I., Knowles B.B. (1979). Controlled synthesis of HBsAg in a differentiated human liver carcinoma-derived cell line. Nature.

[14] Busso N., Chesne C., Delers F., Morel F., Guillouzo A. (1990). Transforming growth-factor-beta (TGF-beta) inhibits albumin synthesis in normal human hepatocytes and in hepatoma HepG2 cells. Biochem. Biophys. Res. Commun..

[15] Meier M., Klein H.H., Kramer J., Drenckhan M., Schütt M. (2007). Calpain inhibition impairs glycogen syntheses in HepG2 hepatoma cells without altering insulin signaling. J. Endocrinol..

[16] Gerets H.H., Tilmant K., Gerin B., Chanteux H., Depelchin B.O., Dhalluin S., Atienzar F.A. (2012). Characterization of primary human hepatocytes, HepG2 cells, and HepaRG cells at the mRNA level and CYP activity in response to inducers and their predictivity for the detection of human hepatotoxins. Cell Biol. Toxicol..

[17] Westerink W.M., Schoonen W.G. (2007). Cytochrome P450 enzyme levels in HepG2 cells and cryopreserved primary human hepatocytes and their induction in HepG2 cells. Toxicol. In Vitro.

[18] Steinbrecht S., Pfeifer N., Herzog N., Katzenberger N., Schulz C., Kammerer S., Küpper J.H. (2020). HepG2-1A2 C2 and C7: Lentivirus vector-mediated stable and functional overexpression of cytochrome P450 1A2 in human hepatoblastoma cells. Toxicol. Lett..

[19] Steinbrecht S., König R., Schmidtke K.U., Herzog N., Scheibner K., Krüger-Genge A., Jung F., Kammerer S., Küpper J.H. (2019). Metabolic activity testing can underestimate acute drug cytotoxicity as revealed by HepG2 cell clones overexpressing cytochrome P450 2C19 and 3A4. Toxicology.

[20] Xuan J., Chen S., Ning B., Tolleson W.H., Guo L. (2016). Development of HepG2-derived cells expressing cytochrome P450s for assessing metabolism-associated drug-induced liver toxicity. Chem. Biol. Interact..

[21] Herzog N., Katzenberger N., Martin F., Schmidtke K.-U., Küpper J.-H. (2015). Generation of cytochrome P450 3A4-overexpressing HepG2 cell clones for standardization of hepatocellular testosterone 6β-hydroxylation activity. J. Cell. Biotechnol..

[22] Yoshitomi S., Ikemoto K., Takahashi J., Miki H., Namba M., Asahi S. (2001). Establishment of the transformants expressing human cytochrome P450 subtypes in HepG2, and their applications on drug metabolism and toxicology. Toxicol. In Vitro.

[23] Gripon P., Rumin S., Urban S., Le Seyec J., Glaise D., Cannie I., Guyomard C., Lucas J., Trepo C., Guguen-Guillouzo C. (2002). Infection of a human hepatoma cell line by hepatitis B virus. Proc. Natl. Acad. Sci. USA.

[24] Jennen D.G., Magkoufopoulou C., Ketelslegers H.B., van Herwijnen M.H., Kleinjans J.C., van Delft J.H. (2010). Comparison of HepG2 and HepaRG by whole-genome gene expression analysis for the purpose of chemical hazard identification. Toxicol. Sci..

[25] Aninat C., Piton A., Glaise D., Le Charpentier T., Langouët S., Morel F., Guguen-Guillouzo C., Guillouzo A. (2006). Expression of cytochromes P450, conjugating enzymes and nuclear receptors in human hepatoma HepaRG cells. Drug Metab. Dispos..

[26] Klein S., Mueller D., Schevchenko V., Noor F. (2014). Long-term maintenance of HepaRG cells in serum-free conditions and application in a repeated dose study. J. Appl. Toxicol..

[27] Jackson J.P., Li L., Chamberlain E.D., Wang H., Ferguson S.S. (2016). Contextualizing Hepatocyte Functionality of Cryopreserved HepaRG Cell Cultures. Drug Metab. Dispos..

[28] Guillouzo A., Corlu A., Aninat C., Glaise D., Morel F., Guguen-Guillouzo C. (2007). The human hepatoma HepaRG cells: A highly differentiated model for studies of liver metabolism and toxicity of xenobiotics. Chem. Biol. Interact..

[29] Zanger U.M., Schwab M. (2013). Cytochrome P450 enzymes in drug metabolism: Regulation of gene expression, enzyme activities, and impact of genetic variation. Pharmacol. Ther..

[30] Goldring C., Antoine D.J., Bonner F., Crozier J., Denning C., Fontana R.J., Hanley N.A., Hay D.C., Ingelman-Sundberg M., Juhila S. (2017). Stem cell–derived models to improve mechanistic understanding and prediction of human drug-induced liver injury. Hepatology.

[31] Kia R., Sison R.L., Heslop J., Kitteringham N.R., Hanley N., Mills J.S., Park B.K., Goldring C.E. (2013). Stem cell-derived hepatocytes as a predictive model for drug-induced liver injury: Are we there yet?. Br. J. Clin. Pharmacol..

[32] Freyer N., Knöspel F., Strahl N., Amini L., Schrade P., Bachmann S., Damm G., Seehofer D., Jacobs F., Monshouwer M. (2016). Hepatic Differentiation of Human Induced Pluripotent Stem Cells in a Perfused Three-Dimensional Multicompartment Bioreactor. Biores. Open Access.

[33] Kang S.J., Lee H.M., Park Y.I., Yi H., Lee H., So B., Song J.Y., Kang H.G. (2016). Chemically induced hepatotoxicity in human stem cell-induced hepatocytes compared with primary hepatocytes and HepG2. Cell Biol. Toxicol..

[34] Baxter M., Withey S., Harrison S., Segeritz C.-P., Zhang F., Atkinson-Dell R., Rowe C., Gerrard D.T., Sison-Young R., Jenkins R. (2015). Phenotypic and functional analyses show stem cell-derived hepatocyte-like cells better mimic fetal rather than adult hepatocytes. J. Hepatol..

[35] Schwartz R.E., Fleming H.E., Khetani S.R., Bhatia S.N. (2014). Pluripotent stem cell-derived hepatocyte-like cells. Biotechnol. Adv..

[36] Takayama K., Morisaki Y., Kuno S., Nagamoto Y., Harada K., Furukawa N., Ohtaka M., Nishimura K., Imagawa K., Sakurai F. (2014). Prediction of interindividual differences in hepatic functions and drug sensitivity by using human iPS-derived hepatocytes. Proc. Natl. Acad. Sci. USA.

[37] Ulvestad M., Nordell P., Asplund A., Rehnström M., Jacobsson S., Holmgren G., Davidson L., Brolén G., Edsbagge J., Björquist P. (2013). Drug metabolizing enzyme and transporter protein profiles of hepatocytes derived from human embryonic and induced pluripotent stem cells. Biochem. Pharmacol..

[38] Gao X., Liu Y. (2017). A transcriptomic study suggesting human iPSC-derived hepatocytes potentially offer a better in vitro model of hepatotoxicity than most hepatoma cell lines. Cell Biol. Toxicol..

[39] Holmgren G., Ulfenborg B., Asplund A., Toet K., Andersson C.X., Hammarstedt A., Hanemaaijer R., Küppers-Munther B., Synnergren J. (2020). Characterization of Human Induced Pluripotent Stem Cell-Derived Hepatocytes with Mature Features and Potential for Modeling Metabolic Diseases. Int. J. Mol. Sci..

[40] Kammerer S., Küpper J.H. (2018). Optimized protocol for induction of cytochrome P450 enzymes 1A2 and 3A4 in human primary-like hepatocyte cell strain HepaFH3 to study in vitro toxicology. Clin. Hemorheol. Microcirc..

[41] Herzog N., Hansen M., Miethbauer S., Schmidtke K.U., Anderer U., Lupp A., Sperling S., Seehofer D., Damm G., Scheibner K. (2016). Primary-like human hepatocytes genetically engineered to obtain proliferation competence display hepatic differentiation characteristics in monolayer and organotypical spheroid cultures. Cell Biol. Int..

[42] Nörenberg A., Heinz S., Scheller K., Hewitt N.J., Braspenning J., Ott M. (2013). Optimization of upcyte^®^ human hepatocytes for the in vitro micronucleus assay. Mutat. Res. Genet. Toxicol. Environ. Mutagenesis.

[43] Burkard A., Dähn C., Heinz S., Zutavern A., Sonntag-Buck V., Maltman D., Przyborski S., Hewitt N.J., Braspenning J. (2012). Generation of proliferating human hepatocytes using upcyte^®^ technology: Characterisation and applications in induction and cytotoxicity assays. Xenobiotica.

[44] Levy G., Bomze D., Heinz S., Ramachandran S.D., Noerenberg A., Cohen M., Shibolet O., Sklan E., Braspenning J., Nahmias Y. (2015). Long-term culture and expansion of primary human hepatocytes. Nat. Biotechnol..

[45] Ramachandran S.D., Vivarès A., Klieber S., Hewitt N.J., Muenst B., Heinz S., Walles H., Braspenning J. (2015). Applicability of second-generation upcyte^®^ human hepatocytes for use in CYP inhibition and induction studies. Pharmacol. Res. Perspect..

[46] Tolosa L., Gómez-Lechón M.J., López S., Guzmán C., Castell J.V., Donato M.T., Jover R. (2016). Human Upcyte Hepatocytes: Characterization of the Hepatic Phenotype and Evaluation for Acute and Long-Term Hepatotoxicity Routine Testing. Toxicol. Sci..

[47] Fu G.B., Huang W.J., Zeng M., Zhou X., Wu H.P., Liu C.C., Wu H., Weng J., Zhang H.D., Cai Y.C. (2019). Expansion and differentiation of human hepatocyte-derived liver progenitor-like cells and their use for the study of hepatotropic pathogens. Cell Res..

[48] Qiao S., Feng S., Wu Z., He T., Ma C., Peng Z., Tian E., Pan G. (2021). Functional Proliferating Human Hepatocytes: In Vitro Hepatocyte Model for Drug Metabolism, Excretion, and Toxicity. Drug Metab. Dispos..

[49] Vinken M., Papeleu P., Snykers S., De Rop E., Henkens T., Chipman J.K., Rogiers V., Vanhaecke T. (2006). Involvement of cell junctions in hepatocyte culture functionality. Crit. Rev. Toxicol..

[50] Riede J., Wollmann B.M., Molden E., Ingelman-Sundberg M. (2021). Primary human hepatocyte spheroids as an in vitro tool for investigating drug compounds with low clearance. Drug Metab. Dispos..

[51] Rose S., Ezan F., Cuvellier M., Bruyère A., Legagneux V., Langouët S., Baffet G. (2021). Generation of proliferating human adult hepatocytes using optimized 3D culture conditions. Sci. Rep..

[52] Mizoi K., Hosono M., Kojima H., Ogihara T. (2020). Establishment of a primary human hepatocyte spheroid system for evaluating metabolic toxicity using dacarbazine under conditions of CYP1A2 induction. Drug Metab. Pharmacokinet..

[53] Bell C.C., Hendriks D.F.G., Moro S.M.L., Ellis E., Walsh J., Renblom A., Fredriksson Puigvert L., Dankers A.C.A., Jacobs F., Snoeys J. (2016). Characterization of primary human hepatocyte spheroids as a model system for drug-induced liver injury, liver function and disease. Sci. Rep..

[54] Messner S., Agarkova I., Moritz W., Kelm J.M. (2013). Multi-cell type human liver microtissues for hepatotoxicity testing. Arch. Toxicol..

[55] Kanebratt K.P., Janefeldt A., Vilén L., Vildhede A., Samuelsson K., Milton L., Björkbom A., Persson M., Leandersson C., Andersson T.B. (2021). Primary Human Hepatocyte Spheroid Model as a 3D In Vitro Platform for Metabolism Studies. J. Pharm. Sci..

[56] Vorrink S.U., Ullah S., Schmidt S., Nandania J., Velagapudi V., Beck O., Ingelman-Sundberg M., Lauschke V.M. (2017). Endogenous and xenobiotic metabolic stability of primary human hepatocytes in long-term 3D spheroid cultures revealed by a combination of targeted and untargeted metabolomics. FASEB J..

[57] Berger B., Donzelli M., Maseneni S., Boess F., Roth A., Krähenbühl S., Haschke M. (2016). Comparison Of Liver Cell Models Using The Basel Phenotyping Cocktail. Front. Pharmacol..

[58] Foster A.J., Chouhan B., Regan S.L., Rollison H., Amberntsson S., Andersson L.C., Srivastava A., Darnell M., Cairns J., Lazic S.E. (2019). Integrated in vitro models for hepatic safety and metabolism: Evaluation of a human Liver-Chip and liver spheroid. Arch. Toxicol..

[59] Bell C.C., Lauschke V.M., Vorrink S.U., Palmgren H., Duffin R., Andersson T.B., Ingelman-Sundberg M. (2017). Transcriptional, Functional, and Mechanistic Comparisons of Stem Cell–Derived Hepatocytes, HepaRG Cells, and Three-Dimensional Human Hepatocyte Spheroids as Predictive In Vitro Systems for Drug-Induced Liver Injury. Drug Metab. Dispos..

[60] Bell C.C., Dankers A.C.A., Lauschke V.M., Sison-Young R., Jenkins R., Rowe C., Goldring C.E., Park K., Regan S.L., Walker T. (2018). Comparison of Hepatic 2D Sandwich Cultures and 3D Spheroids for Long-term Toxicity Applications: A Multicenter Study. Toxicol. Sci..

[61] Vorrink S.U., Zhou Y., Ingelman-Sundberg M., Lauschke V.M. (2018). Prediction of Drug-Induced Hepatotoxicity Using Long-Term Stable Primary Hepatic 3D Spheroid Cultures in Chemically Defined Conditions. Toxicol. Sci. Off. J. Soc. Toxicol..

[62] Hendriks D.F., Fredriksson Puigvert L., Messner S., Mortiz W., Ingelman-Sundberg M. (2016). Hepatic 3D spheroid models for the detection and study of compounds with cholestatic liability. Sci. Rep..

[63] Kozyra M., Johansson I., Nordling Å., Ullah S., Lauschke V.M., Ingelman-Sundberg M. (2018). Human hepatic 3D spheroids as a model for steatosis and insulin resistance. Sci. Rep..

[64] Rubiano A., Indapurkar A., Yokosawa R., Miedzik A., Rosenzweig B., Arefin A., Moulin C.M., Dame K., Hartman N., Volpe D.A. (2021). Characterizing the reproducibility in using a liver microphysiological system for assaying drug toxicity, metabolism, and accumulation. Clin. Transl. Sci..

[65] Kukla D.A., Crampton A.L., Wood D.K., Khetani S.R. (2020). Microscale Collagen and Fibroblast Interactions Enhance Primary Human Hepatocyte Functions in Three-Dimensional Models. Gene Expr..

[66] Messner S., Fredriksson L., Lauschke V.M., Roessger K., Escher C., Bober M., Kelm J.M., Ingelman-Sundberg M., Moritz W. (2018). Transcriptomic, Proteomic, and Functional Long-Term Characterization of Multicellular Three-Dimensional Human Liver Microtissues. Appl. In Vitro Toxicol..

[67] Baze A., Parmentier C., Hendriks D.F., Hurrell T., Heyd B., Bachellier P., Schuster C., Ingelman-Sundberg M., Richert L. (2018). Three-dimensional spheroid primary human hepatocytes in monoculture and coculture with nonparenchymal cells. Tissue Eng. Part C Methods.

[68] Bell C.C., Chouhan B., Andersson L.C., Andersson H., Dear J.W., Williams D.P., Söderberg M. (2020). Functionality of primary hepatic non-parenchymal cells in a 3D spheroid model and contribution to acetaminophen hepatotoxicity. Arch. Toxicol..

[69] Li F., Cao L., Parikh S., Zuo R. (2020). Three-dimensional spheroids with primary human liver cells and differential roles of kupffer cells in drug-induced liver injury. J. Pharm. Sci..

[70] Proctor W.R., Foster A.J., Vogt J., Summers C., Middleton B., Pilling M.A., Shienson D., Kijanska M., Ströbel S., Kelm J.M. (2017). Utility of spherical human liver microtissues for prediction of clinical drug-induced liver injury. Arch. Toxicol..

[71] Nguyen D.G., Funk J., Robbins J.B., Crogan-Grundy C., Presnell S.C., Singer T., Roth A.B. (2016). Bioprinted 3D primary liver tissues allow assessment of organ-level response to clinical drug induced toxicity in vitro. PLoS ONE.

[72] Tostões R.M., Leite S.B., Serra M., Jensen J., Björquist P., Carrondo M.J.T., Brito C., Alves P.M. (2012). Human liver cell spheroids in extended perfusion bioreactor culture for repeated-dose drug testing. Hepatology.

[73] Rebelo S.P., Costa R., Silva M.M., Marcelino P., Brito C., Alves P.M. (2017). Three-dimensional co-culture of human hepatocytes and mesenchymal stem cells: Improved functionality in long-term bioreactor cultures. J. Tissue Eng. Regen. Med..

[74] Štampar M., Breznik B., Filipič M., Žegura B. (2020). Characterization of In Vitro 3D Cell Model Developed from Human Hepatocellular Carcinoma (HepG2) Cell Line. Cells.

[75] Štampar M., Tomc J., Filipič M., Žegura B. (2019). Development of in vitro 3D cell model from hepatocellular carcinoma (HepG2) cell line and its application for genotoxicity testing. Arch. Toxicol..

[76] Gaskell H., Sharma P., Colley H.E., Murdoch C., Williams D.P., Webb S.D. (2016). Characterization of a functional C3A liver spheroid model. Toxicol. Res..

[77] Takahashi Y., Hori Y., Yamamoto T., Urashima T., Ohara Y., Tanaka H. (2015). 3D spheroid cultures improve the metabolic gene expression profiles of HepaRG cells. Biosci. Rep..

[78] Chang T.T., Hughes-Fulford M. (2009). Monolayer and spheroid culture of human liver hepatocellular carcinoma cell line cells demonstrate distinct global gene expression patterns and functional phenotypes. Tissue Eng. Part A.

[79] Elje E., Hesler M., Rundén-Pran E., Mann P., Mariussen E., Wagner S., Dusinska M., Kohl Y. (2019). The comet assay applied to HepG2 liver spheroids. Mutat. Res./Genet. Toxicol. Environ. Mutagenesis.

[80] Štampar M., Sedighi Frandsen H., Rogowska-Wrzesinska A., Wrzesinski K., Filipič M., Žegura B. (2021). Hepatocellular carcinoma (HepG2/C3A) cell-based 3D model for genotoxicity testing of chemicals. Sci. Total Environ..

[81] Sasaki K., Akagi T., Asaoka T., Eguchi H., Fukuda Y., Iwagami Y., Yamada D., Noda T., Wada H., Gotoh K. (2017). Construction of three-dimensional vascularized functional human liver tissue using a layer-by-layer cell coating technique. Biomaterials.

[82] Mori N., Kida Y.S. (2020). Expression of genes involved in drug metabolism differs between perfusable 3D liver tissue and conventional 2D-cultured hepatocellular carcinoma cells. FEBS Open Bio.

[83] Kang H.K., Sarsenova M., Kim D.H., Kim M.S., Lee J.Y., Sung E.A., Kook M.G., Kim N.G., Choi S.W., Ogay V. (2021). Establishing a 3D In Vitro Hepatic Model Mimicking Physiologically Relevant to In Vivo State. Cells.

[84] Gori M., Giannitelli S.M., Torre M., Mozetic P., Abbruzzese F., Trombetta M., Traversa E., Moroni L., Rainer A. (2020). Biofabrication of Hepatic Constructs by 3D Bioprinting of a Cell-Laden Thermogel: An Effective Tool to Assess Drug-Induced Hepatotoxic Response. Adv. Healthc. Mater..

[85] Taymour R., Kilian D., Ahlfeld T., Gelinsky M., Lode A. (2021). 3D bioprinting of hepatocytes: Core-shell structured co-cultures with fibroblasts for enhanced functionality. Sci. Rep..

[86] Mueller D., Krämer L., Hoffmann E., Klein S., Noor F. (2014). 3D organotypic HepaRG cultures as in vitro model for acute and repeated dose toxicity studies. Toxicology In Vitro.

[87] Cuvellier M., Ezan F., Oliveira H., Rose S., Fricain J.C., Langouët S., Legagneux V., Baffet G. (2021). 3D culture of HepaRG cells in GelMa and its application to bioprinting of a multicellular hepatic model. Biomaterials.

[88] Ott L.M., Ramachandran K., Stehno-Bittel L. (2017). An Automated Multiplexed Hepatotoxicity and CYP Induction Assay Using HepaRG Cells in 2D and 3D. SLAS DISCOVERY Adv. Sci. Drug Discov..

[89] Wang Z., Luo X., Anene-Nzelu C., Yu Y., Hong X., Singh N.H., Xia L., Liu S., Yu H. (2015). HepaRG culture in tethered spheroids as an in vitro three-dimensional model for drug safety screening. J. Appl. Toxicol..

[90] Gunness P., Mueller D., Shevchenko V., Heinzle E., Ingelman-Sundberg M., Noor F. (2013). 3D Organotypic Cultures of Human HepaRG Cells: A Tool for In Vitro Toxicity Studies. Toxicol. Sci..

[91] Leite S.B., Wilk-Zasadna I., Zaldivar J.M., Airola E., Reis-Fernandes M.A., Mennecozzi M., Guguen-Guillouzo C., Chesne C., Guillou C., Alves P.M. (2012). Three-Dimensional HepaRG Model As An Attractive Tool for Toxicity Testing. Toxicol. Sci..

[92] Nelson L.J., Morgan K., Treskes P., Samuel K., Henderson C.J., LeBled C., Homer N., Grant M.H., Hayes P.C., Plevris J.N. (2017). Human Hepatic HepaRG Cells Maintain an Organotypic Phenotype with High Intrinsic CYP450 Activity/Metabolism and Significantly Outperform Standard HepG2/C3A Cells for Pharmaceutical and Therapeutic Applications. Basic Clin. Pharmacol. Toxicol..

[93] Mandon M., Huet S., Dubreil E., Fessard V., Le Hégarat L. (2019). Three-dimensional HepaRG spheroids as a liver model to study human genotoxicity in vitro with the single cell gel electrophoresis assay. Sci. Rep..

[94] Zhang C., Zhang Q., Li J., Yu L., Li F., Li W., Li Y., Peng H., Zhao J., Carmichael P.L. (2020). Integration of in vitro data from three dimensionally cultured HepaRG cells and physiologically based pharmacokinetic modeling for assessment of acetaminophen hepatotoxicity. Regul. Toxicol. Pharmacol..

[95] Basharat A., Rollison H.E., Williams D.P., Ivanov D.P. (2020). HepG2 (C3A) spheroids show higher sensitivity compared to HepaRG spheroids for drug-induced liver injury (DILI). Toxicol. Appl. Pharmacol..

[96] Weltin A., Hammer S., Noor F., Kaminski Y., Kieninger J., Urban G.A. (2017). Accessing 3D microtissue metabolism: Lactate and oxygen monitoring in hepatocyte spheroids. Biosens. Bioelectron..

[97] Leite S.B., Roosens T., El Taghdouini A., Mannaerts I., Smout A.J., Najimi M., Sokal E., Noor F., Chesne C., van Grunsven L.A. (2016). Novel human hepatic organoid model enables testing of drug-induced liver fibrosis in vitro. Biomaterials.

[98] Rashidi H., Luu N.-T., Alwahsh S.M., Ginai M., Alhaque S., Dong H., Tomaz R.A., Vernay B., Vigneswara V., Hallett J.M. (2018). 3D human liver tissue from pluripotent stem cells displays stable phenotype in vitro and supports compromised liver function in vivo. Arch. Toxicol..

[99] Meier F., Freyer N., Brzeszczynska J., Knöspel F., Armstrong L., Lako M., Greuel S., Damm G., Ludwig-Schwellinger E., Deschl U. (2017). Hepatic differentiation of human iPSCs in different 3D models: A comparative study. Int. J. Mol. Med..

[100] Takayama K., Kawabata K., Nagamoto Y., Kishimoto K., Tashiro K., Sakurai F., Tachibana M., Kanda K., Hayakawa T., Furue M.K. (2013). 3D spheroid culture of hESC/hiPSC-derived hepatocyte-like cells for drug toxicity testing. Biomaterials.

[101] Lee G., Kim H., Park J.Y., Kim G., Han J., Chung S., Yang J.H., Jeon J.S., Woo D.H., Han C. (2021). Generation of uniform liver spheroids from human pluripotent stem cells for imaging-based drug toxicity analysis. Biomaterials.

[102] Qosa H., Ribeiro A.J.S., Hartman N.R., Volpe D.A. (2021). Characterization of a commercially available line of iPSC hepatocytes as models of hepatocyte function and toxicity for regulatory purposes. J. Pharmacol. Toxicol. Methods.

[103] Goulart E., de Caires-Junior L.C., Telles-Silva K.A., Araujo B.H.S., Rocco S.A., Sforca M., de Sousa I.L., Kobayashi G.S., Musso C.M., Assoni A.F. (2019). 3D bioprinting of liver spheroids derived from human induced pluripotent stem cells sustain liver function and viability in vitro. Biofabrication.

[104] Ardalani H., Sengupta S., Harms V., Vickerman V., Thomson J.A., Murphy W.L. (2019). 3-D culture and endothelial cells improve maturity of human pluripotent stem cell-derived hepatocytes. Acta Biomater..

[105] Sirenko O., Hancock M.K., Hesley J., Hong D., Cohen A., Gentry J., Carlson C.B., Mann D.A. (2016). Phenotypic characterization of toxic compound effects on liver spheroids derived from iPSC using confocal imaging and three-dimensional image analysis. Assay Drug Dev. Technol..

[106] Wang Z., Li W., Jing H., Ding M., Fu G., Yuan T., Huang W., Dai M., Tang D., Zeng M. (2019). Generation of hepatic spheroids using human hepatocyte-derived liver progenitor-like cells for hepatotoxicity screening. Theranostics.

[107] Thompson W.L., Takebe T. (2020). Generation of multi-cellular human liver organoids from pluripotent stem cells. Methods Cell Biol..

[108] Hu H., Gehart H., Artegiani B., LÖpez-Iglesias C., Dekkers F., Basak O., van Es J., Chuva de Sousa Lopes S.M., Begthel H., Korving J. (2018). Long-Term Expansion of Functional Mouse and Human Hepatocytes as 3D Organoids. Cell.

[109] Huch M., Gehart H., Van Boxtel R., Hamer K., Blokzijl F., Verstegen M.M., Ellis E., Van Wenum M., Fuchs S.A., de Ligt J. (2015). Long-term culture of genome-stable bipotent stem cells from adult human liver. Cell.

[110] Mun S.J., Hong Y.H., Ahn H.S., Ryu J.S., Chung K.S., Son M.J. (2020). Long-Term Expansion of Functional Human Pluripotent Stem Cell-Derived Hepatic Organoids. Int. J. Stem Cells.

[111] Ramli M.N.B., Lim Y.S., Koe C.T., Demircioglu D., Tng W., Gonzales K.A.U., Tan C.P., Szczerbinska I., Liang H., Soe E.L. (2020). Human pluripotent stem cell-derived organoids as models of liver disease. Gastroenterology.

[112] Pettinato G., Lehoux S., Ramanathan R., Salem M.M., He L.X., Muse O., Flaumenhaft R., Thompson M.T., Rouse E.A., Cummings R.D. (2019). Generation of fully functional hepatocyte-like organoids from human induced pluripotent stem cells mixed with Endothelial Cells. Sci. Rep..

[113] Pless G., Steffen I., Zeilinger K., Sauer I.M., Katenz E., Kehr D.C., Roth S., Mieder T., Schwartlander R., Müller C. (2006). Evaluation of Primary Human Liver Cells in Bioreactor Cultures for Extracorporeal Liver Support on the Basis of Urea Production. Artif. Organs.

[114] Gerlach J.C., Mutig K., Sauer I.M., Schrade P., Efimova E., Mieder T., Naumann G., Grunwald A., Pless G., Mas A. (2003). Use of primary human liver cells originating from discarded grafts in a bioreactor for liver support therapy and the prospects of culturing adult liver stem cells in bioreactors: A morphologic study. Transplantation.

[115] Lee-Montiel F.T., George S.M., Gough A.H., Sharma A.D., Wu J., DeBiasio R., Vernetti L.A., Taylor D.L. (2017). Control of oxygen tension recapitulates zone-specific functions in human liver microphysiology systems. Exp. Biol. Med..

[116] Gehre C., Flechner M., Kammerer S., Küpper J.H., Coleman C.D., Püschel G.P., Uhlig K., Duschl C. (2020). Real time monitoring of oxygen uptake of hepatocytes in a microreactor using optical microsensors. Sci. Rep..

[117] Peel S., Corrigan A.M., Ehrhardt B., Jang K.-J., Caetano-Pinto P., Boeckeler M., Rubins J.E., Kodella K., Petropolis D.B., Ronxhi J. (2019). Introducing an automated high content confocal imaging approach for Organs-on-Chips. Lab Chip.

[118] Bavli D., Prill S., Ezra E., Levy G., Cohen M., Vinken M., Vanfleteren J., Jaeger M., Nahmias Y. (2016). Real-time monitoring of metabolic function in liver-on-chip microdevices tracks the dynamics of mitochondrial dysfunction. Proc. Natl. Acad. Sci. USA.

[119] Prill S., Bavli D., Levy G., Ezra E., Schmälzlin E., Jaeger M.S., Schwarz M., Duschl C., Cohen M., Nahmias Y. (2016). Real-time monitoring of oxygen uptake in hepatic bioreactor shows CYP450-independent mitochondrial toxicity of acetaminophen and amiodarone. Arch. Toxicol..

[120] Hoffmann S.A., Müller-Vieira U., Biemel K., Knobeloch D., Heydel S., Lübberstedt M., Nüssler A.K., Andersson T.B., Gerlach J.C., Zeilinger K. (2012). Analysis of drug metabolism activities in a miniaturized liver cell bioreactor for use in pharmacological studies. Biotechnol. Bioeng..

[121] Zeilinger K., Schreiter T., Darnell M., Söderdahl T., Lübberstedt M., Dillner B., Knobeloch D., Nüssler A.K., Gerlach J.C., Andersson T.B. (2011). Scaling down of a clinical three-dimensional perfusion multicompartment hollow fiber liver bioreactor developed for extracorporeal liver support to an analytical scale device useful for hepatic pharmacological in vitro studies. Tissue Eng. Part C Methods.

[122] Lübberstedt M., Müller-Vieira U., Biemel K.M., Darnell M., Hoffmann S.A., Knöspel F., Wönne E.C., Knobeloch D., Nüssler A.K., Gerlach J.C. (2015). Serum-free culture of primary human hepatocytes in a miniaturized hollow-fibre membrane bioreactor for pharmacological in vitro studies. J. Tissue Eng. Regen. Med..

[123] Jang K.-J., Otieno M.A., Ronxhi J., Lim H.-K., Ewart L., Kodella K.R., Petropolis D.B., Kulkarni G., Rubins J.E., Conegliano D. (2019). Reproducing human and cross-species drug toxicities using a Liver-Chip. Sci. Transl. Med..

[124] Du Y., Li N., Yang H., Luo C., Gong Y., Tong C., Gao Y., Lü S., Long M. (2017). Mimicking liver sinusoidal structures and functions using a 3D-configured microfluidic chip. Lab Chip.

[125] Choi Y.Y., Seok J.I., Kim D.S. (2019). Flow-Based Three-Dimensional Co-Culture Model for Long-Term Hepatotoxicity Prediction. Micromachines.

[126] Knowlton S., Tasoglu S. (2016). A bioprinted liver-on-a-chip for drug screening applications. Trends Biotechnol..

[127] Ma L.-D., Wang Y.-T., Wang J.-R., Wu J.-L., Meng X.-S., Hu P., Mu X., Liang Q.-L., Luo G.-A. (2018). Design and fabrication of a liver-on-a-chip platform for convenient, highly efficient, and safe in situ perfusion culture of 3D hepatic spheroids. Lab Chip.

[128] Corrado B., De Gregorio V., Imparato G., Attanasio C., Urciuolo F., Netti P.A. (2019). A three-dimensional microfluidized liver system to assess hepatic drug metabolism and hepatotoxicity. Biotechnol. Bioeng..

[129] Meng Q., Wang Y., Li Y., Shen C. (2021). Hydrogel microfluidic-based liver-on-a-chip: Mimicking the mass transfer and structural features of liver. Biotechnol. Bioeng..

[130] Hong G., Kim J., Oh H., Yun S., Kim C.M., Jeong Y.M., Yun W.S., Shim J.H., Jang I., Kim C.Y. (2021). Production of Multiple Cell-Laden Microtissue Spheroids with a Biomimetic Hepatic-Lobule-Like Structure. Adv. Mater..

[131] Ulvestad M., Darnell M., Molden E., Ellis E., Åsberg A., Andersson T.B. (2012). Evaluation of organic anion-transporting polypeptide 1B1 and CYP3A4 activities in primary human hepatocytes and HepaRG cells cultured in a dynamic three-dimensional bioreactor system. J. Pharmacol. Exp. Ther..

[132] Boul M., Benzoubir N., Messina A., Ghasemi R., Mosbah I.B., Duclos-Vallée J.C., Dubart-Kupperschmitt A., Le Pioufle B. (2021). A versatile microfluidic tool for the 3D culture of HepaRG cells seeded at various stages of differentiation. Sci. Rep..

[133] Jang M., Kleber A., Ruckelshausen T., Betzholz R., Manz A. (2019). Differentiation of the human liver progenitor cell line (HepaRG) on a microfluidic-based biochip. J. Tissue Eng. Regen. Med..

[134] Rennert K., Steinborn S., Gröger M., Ungerböck B., Jank A.-M., Ehgartner J., Nietzsche S., Dinger J., Kiehntopf M., Funke H. (2015). A microfluidically perfused three dimensional human liver model. Biomaterials.

[135] Wang Y., Wang H., Deng P., Chen W., Guo Y., Tao T., Qin J. (2018). In situ differentiation and generation of functional liver organoids from human iPSCs in a 3D perfusable chip system. Lab Chip.

[136] Bircsak K.M., DeBiasio R., Miedel M., Alsebahi A., Reddinger R., Saleh A., Shun T., Vernetti L.A., Gough A. (2021). A 3D microfluidic liver model for high throughput compound toxicity screening in the OrganoPlate^®^. Toxicology.

